# Can Diaphragm Dysfunction Be Reliably Evaluated with Pocket-Sized Ultrasound Devices in Intensive Care Unit?

**DOI:** 10.1155/2018/5192647

**Published:** 2018-04-01

**Authors:** Gul Gursel, Kamil Inci, Zenfira Alasgarova

**Affiliations:** ^1^Department of Pulmonary Critical Care Medicine, Gazi University School of Medicine, Ankara, Turkey; ^2^Division of Critical Care Medicine, Department of Internal Medicine, Gazi University School of Medicine, Ankara, Turkey

## Abstract

**Background:**

Diaphragm dysfunction (DD) is frequently seen in critically ill patients, and ultrasound could be a useful tool to detect it and to predict extubation success or failure in mechanically ventilated patients. Besides, it would also be useful in differential diagnosis of dyspnea and respiratory failure. The aim of this study is to evaluate usefulness and accuracy of pocket-sized ultrasound devices (PSDs) in assessment of DD in intensive care unit (ICU) patients in comparison with standard ultrasound devices (SD).

**Methods:**

In this prospective observational study, we compared the performance of PSD and SD in visualization of diaphragm, detection of paradoxical movement, measurement of tidal and maximal thickness, tidal and maximal excursion, and calculation of thickening fraction (TF) of the diaphragm. We used Bland and Altman test for agreement and bias analysis and intraclass correlation analysis to evaluate interobserver variability.

**Results:**

Thirty-nine patients were included in the study. In 93% of the patients, diaphragm was visualized with PSD. There was very good agreement between the measurements of the devices, and there was no proportional bias in the measurements of tidal inspiratory and expiratory thickness, tidal TF, tidal excursion, and maximal inspiratory thickness. In interobserver reliability analysis of all measurements for both devices, ICC coefficients were higher than 0.8. Total diaphragm examination times of the devices were similar (*p* > 0.05).

**Conclusion:**

These results suggest that PSD can be useful in ICU patients for evaluating DD. But further studies are required to determine the exact place of these devices in evaluation of DD in ICU patients.

## 1. Introduction

Diaphragm dysfunction (DD) occurs in about 60% of intensive care unit (ICU) patients because of risk factors such as sepsis, disease severity, and mechanical ventilation [1]. Not only medical but also surgical ICU patients are at risk because of postoperative DD or trauma. Diaphragm ultrasound (DUS) could be a useful and accurate tool to detect DD [1–4] in critically ill patients, to predict extubation success or failure [5–9] and to assess and monitorize diaphragm weakness in mechanically ventilated patients [9, 10] according to recent literature. Besides, it would be a useful tool for differential diagnosis of dyspnea and respiratory failure in ICU patients. Diaphragm function can be assessed by measuring its thickness, thickening fraction (TF), and inspiratory excursion. Thickness and TF are measured by linear probe at the zone of apposition; excursion is measured in the subcostal area with sector or convex probes. Both M (MM) and B (2D) mode can be used to do these measurements [11, 12]. There are lots of studies in the literature performed by standard ultrasound machines (SD). But in general, they are not affordable for some centers, it is not easy to learn how to use them, and they are not practical for busy environments like ICUs. On the other hand, clinicians can also have good quality images with smaller and more affordable pocket-sized ultrasound devices They also allow for rapid and instant assessment, which are extremely important for critical care physicians. They significantly reduce patient's waiting time and improve clinician's workflow. Furthermore, the cost of PSDs is much lower than that of standard ones. There are number of studies assessing the clinical effectiveness of PSDs in certain clinical settings, particularly in cardiology [13–16]. They have suggested that these devices may be safely used to enhance the diagnostic accuracy of cardiovascular or abdominal examination and proposed their use in various clinical settings such as outpatient clinics and Emergency Departments (EDs) [17]. Furthermore, since their introduction to clinical practice, several studies have compared these new PSD with the standard, high quality ultrasound devices and found a high level of correlation in terms of basic diagnostic accuracy [18, 19]. However, its applicability for imaging and measurement of the diaphragm has not been studied yet. In this study, we compared the quality of images and measurements obtained from a PSD and that of a SD in evaluation of diaphragm function in ICU patients.

## 2. Materials and Methods

### 2.1. Patient Population

This prospective observational study was carried out in a University Hospital medical ICU. All patients admitted to the ICU were included in the study consequently. The study protocol was approved by the ethics committee of our institution (28.12.2015/166), and written informed consent was obtained from the patient or his/her next of kin.

### 2.2. Measurement Methods

Ultrasonographic examinations were completed in the same session using the ultrasound machines in a randomized order. Examinations were carried out according to a standardized protocol by two intensivists experienced in ultrasonography.

### 2.3. Devices

We used a VScan (with dual probe) device by GE Systems as PSD and Vivid-Q as a SD (full range of standard modalities and measurements: MM, 2D, PW, CW, Colour, TVI, and TEE). Diaphragm thickness (DT) was measured with linear probes of the machines (7–13 MHz in Vivid-Q and 4–8 MHz in VScan), and excursion was measured with phase array probes (3.5 MHz in VQ and 1.7–3.8 MHz in VScan). Since PSD does not have an MM, we measured DT with B mode in both devices. DE was measured by MM in SD device and by B mode in PSD. To measure DE with PSD, the deepness scale on the screen of the PSD was used. We compared the measurements of images obtained by the SD and the PSD in the evaluation of DT and DE.

### 2.4. DUS Measurements

**Figure 1:**
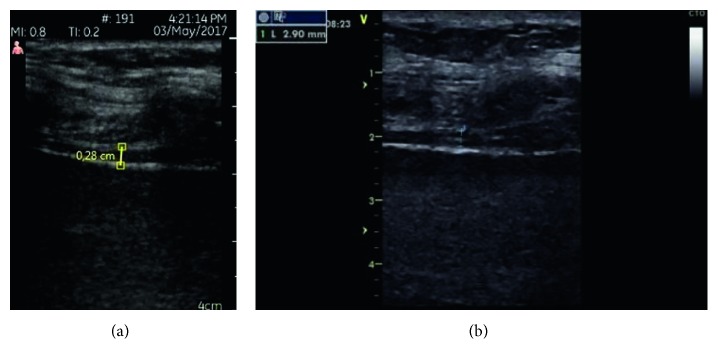
(a) Expiratory diaphragm thickness measured by PSD. (b) Expiratory diaphragm thickness measured by SD.

All measurements were performed on the right side of the patients while patients were in semirecumbent position. Diaphragm examinations were performed while patient is deconnected from the ventilator for intubated patients and patients receiving noninvasive ventilation. For evaluating diaphragmatic thickness (DT) parameters, diaphragm was visualized at the zone of apposition by placing the probe perpendicular to the chest wall, in the eighth or tenth intercostal space, between the anterior axillary and the midaxillary lines. The diaphragm was imaged as a structure formed of two echoic lines (the diaphragmatic pleura and the peritoneal membrane) and a hypoechoic structure between them [12, 20] (Figures 1(a) and 1(b)). Several images of diaphragm were captured during quiet tidal breathing and maximum inspiration. On each B mode image, diaphragm thickness (DT) was measured from middle of the pleural line to middle of the peritoneal line during tidal and maximal inspirations and also during expiration. Measurements were repeated on three consecutive respiratory cycles, and the mean of three measurements was recorded. Then, thickening fraction of the diaphragm (TF) was calculated as a percentage from the following formula:

(1) $$\begin{array}{c} \text{TF}=\left(\frac{\left({\text{TD}}_{\max}-{\text{TD}}_{\min}\right)}{{\text{TD}}_{\min}}\right)\times 100. \end{array}$$

All DE examinations were performed in supine position. The probe was placed below the right subcostal margin in the midclavicular line and moved till better appearance of the posterior third of the right diaphragm. Diaphragm movements were recorded in MM during quiet breathing and deep breathing. The distance between maximal and minimal echogenic lines was measured on frozen images from the M mode tracings in SD. In the PSD, deepness scale of the device was used for the measurements of DE during tidal and maximal breathing. The ultrasound images were stored digitally during the examinations on both instruments. Three different images were recorded, and mean of these 3 measurements was calculated. We also compared the time necessary to complete the ultrasonographic assessment of the diaphragm with both devices. Ultrasound was performed by two intensivists experienced in diaphragm ultrasound. To assess the reproducibility of DT measurements, we performed ten assessments on our ten different patients. The images were analyzed separately by two ultrasonographers to assess interobserver reproducibility.

#### 2.4.1. Definitions of Diaphragm Dysfunction (DD)

TF ≤ 20% and/or tidal DE less than 10 mm [20, 21].

## 3. Statistical Analysis

The number of necessary patients was 35 according to power analyses for significance level of 0.05. Continuous variables were described as mean ± standard deviation or median (interquartile range) depending on whether distribution was normal or not. *p* values lower than 0.05 were considered as statistically significant. Bland–Altman analysis was used to assess if there is agreement and any significant proportional bias between the measurements. Interobserver reliability of diaphragmatic measurements was assessed using the intraclass correlation test (ICC). Intraclass correlation greater than 0.7 was taken to indicate a strong correlation. The evaluation was carried out with SPSS statistical programme.

## 4. Results

**Figure 2:**
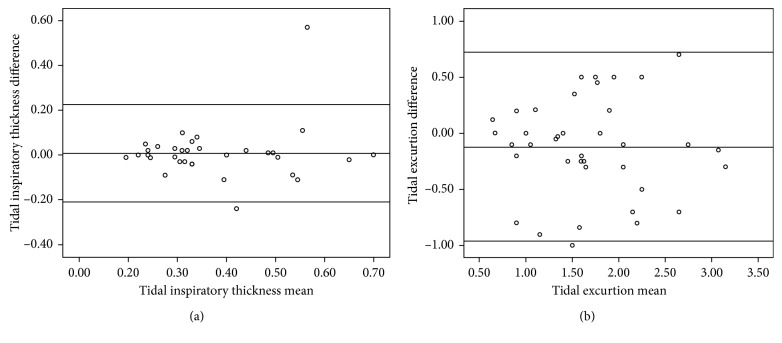
(a) Bland–Altman graphics of inspiratory diaphragm thickness. (b) Bland–Altman graphics of tidal diaphragm excursion.

Thirty-nine patients were included in the study. Demographic features and diagnostic properties of the patients are given in Table 1. There were no surgical patients in the study. Nineteen (48%) of the patients were receiving mechanical ventilation therapy, 10 (26%) were under noninvasive ventilation therapy, and 10 (26%) of them were not receiving any of them. We visualized diaphragm in 36 patients with PSD. Three patients whose diaphragm could not be visualized were obese and had subcutaneous edema. In 4 patients, we detected paradoxical breathing pattern with both devices. In Bland–Altman analyses, there were very good agreement between the measurements of the devices and there was no proportional bias in the measurements of tidal inspiratory and expiratory thickness, tidal TF, tidal DE, and maximal inspiratory thickness. Figures 2(a) and 2(b) show Bland–Altman graphics of tidal inspiratory thickness and tidal excursion of the diaphragm, respectively. There was no agreement between measurements of the devices in only maximal diaphragm excursion measurement in the Bland–Altman test (*p* > 0.05) (Table 2). There was also proportional bias in this measurement. When we considered DD as TF less than 20% and/or diaphragm tidal excursion less than 10 mm, TF measured ≤ 20% with SD in 23% of the patients and with PSD in 13% of the patients. In 18% and 27% of the patients, SD and PSD measured diaphragm tidal excursion less than 10 mm, respectively. According to our definition, SD detected DD in 33% and PSD detected DD in 36% of our patients. Mean time for overall examination with both devices was similar (PSD: 14 ± 4 min, SD: 15 ± 4 min, *p*=0.120). In interobserver reliability analysis of all measurements for both devices, ICC coefficients were higher than 0.80.

## 5. Discussion

Diaphragm dysfunction may play an important role in etiology of difficult weaning, dyspnea, and respiratory failure, and routine ultrasonographic examination of diaphragm may give important information about its function. DD as a result of phrenic nerve paralysis due to trauma, cardiothoracic, or neck surgery can also be detected by DUS [20]. Additionally, it would be useful to monitor diaphragm during pulmonary rehabilitation by physiotherapists. Evaluation of diaphragm function with standard ultrasound machines was reported extensively in the literature. On the other hand, evaluating diaphragm routinely may not require sophisticated, expensive machines which are difficult to learn. In recent years, widespread use of ultrasound in all areas of medicine and development in ultrasound machine technology resulted in production of pocket-sized ultrasound devices. The utility of handheld ultrasound devices has been reported in several medical professions, mainly in point-of-care setting by cardiologists, internists, and emergency physicians [22–26]. They investigated these practical devices in examination of heart, abdomen, urogenital system, dyspnea etiology, and also FAST examination in ED and found that PSDs could visualize all these systems as good as standard ultrasound machines with acceptable intra-/interoperator reproducibility [14, 16]. These studies reported different performance results according to their aims. Some of them demonstrated that these devices are not only easy to operate but in appropriate conditions may provide diagnostic yield similar to that of standard devices, with regards to basic parameters [18, 19]. On the other hand, Stock et al. found that organ size measurements are substantially smaller with the portable instrument than SD, which can make diagnosis less reliable. For example, this may lead to kidneys being wrongly assessed as atrophic or borderline hepatomegaly, and splenomegaly can go unnoticed depending on the results of their study performed in an internal medicine department [18]. In their conclusion, they underscored the limitations of the devices saying that there may be clinical roles for distinct clinical questions such as detection of ascites or pleural effusion when used by experienced examiners. However, sensitivity in detecting multiple pathologies is not comparable to SDs. In another study, comparing SD with PSD in abdominal ultrasonographic examination, PSD was found to be suitable for detecting number of pathologies, such as hydronephrosis, gallstones, intrahepatic ductal stones and dilatation, intra-abdominal collection, major vessel abnormality such as abdominal aortic aneurysm, fluid collection (pleural effusion or ascites), and urogenital examination. However, they recommended examination with standard ultrasound machine when solid organ pathologies such as parenchymal disease and space-occupying lesions are clinically suspected [17]. Lavi et al. compared PSD with SD in urological examination, and they reported that handheld device can be used in evaluating the upper and lower urinary tract with the exception of renal masses. According to their experience, PSD is not sufficient for evaluating focal renal lesions [19]. Sforza et al. aimed to test the usefulness and accuracy of lung ultrasound alone or combined with ultrasound of heart and inferior vena cava (IVC) using a PSD for differential diagnosis of acute dyspnea [20]. Overall, the integrated lung-heart-inferior vena cava ultrasound examination improved the accuracy of LUS alone, by maximizing specificity and allowing to capture different types of heart failure. They found PSD is useful and efficient, and it reduced the necessary time in differential diagnosis of acute dyspnea in ED. They concluded that integrated evaluation of heart-lung and IVC with PSD makes a useful extension of clinical examination, and PSDs can be readily available at the bedside or in an ambulance, which requires only few minutes to apply; additionally, it also has a reliable diagnostic discriminant ability in the setting of acute dyspnea. They did not evaluate diaphragms' of the patients in their study. Baugher and coworkers studied PSD in FAST examination in the ED in trauma patients and found that the image scores obtained with a handheld ultrasound device were lower than those from a standard system commonly used in the ED. On the other hand, when the goal is to confirm the presence or absence of fluid, there was a trend towards more agreement between the systems [22]. In cardiology, PSDs place is defined in a consensus report as complement to physical examination in coronary care unit and ICU, fast initial screening in ED, cardiologic counselling, first cardiac evaluation in ambulances, screening, training, and quantification of extravascular lung water [23]. On the contrary, there is no data yet about using these devices in evaluation of diaphragmatic function in the literature. In this study, we wanted to evaluate the value of PSDs' in comparison with standard ones in the meaning of accuracy of measurements and duration of imaging and measurements. At the end of the study, we saw that, with a PSD, diaphragm can be visualized as good as standard ultrasound devices. In 90% of the patients, diaphragm image acquisition was achieved, and paradox movement was detected as good as SD. Despite the lack of M mode, diaphragm motion could be quantified close to standard machines in PSD. Additionally, there were very good agreement between the measurements of two devices, and there was no bias in measurements of tidal inspiratory and expiratory thickness, tidal TF, tidal DE, and maximal inspiratory thickness according to Bland–Altman test. At first sight, it may seem to be surprising after summarized PSDs performance in abdominal imaging above. But actually, it is not a surprise because, despite the diaphragm is an abdominal structure, its thickness was measured at the zone of apposition which places very close to skin (as if superficial structure). Probably for this reason, we found DT measurements of both devices very close to each other also including TF. Therefore, PSD can be useful in measuring the thickness of diaphragm in ICU setting. When we compared DE measurements of these devices, we saw that correlation and agreement were worse than thickness measurements. Furthermore, there were significant bias and no agreement between the measurements of maximal excursion. This may be explained by the lack of MM or possible inadequate visualization of deep abdominal structures with these portable devices. Another possible explanation is probability of variations in deepness of each breath during the measurement of maximal DE while the patient is breathing deeply. Additionally, measurements of maximal DE with both devices were not carried out at the same time. There were few minutes of differences between the measurements made with both devices, and this may also lead to measurements to be done at different breath deepnesses. To overcome this problem, placing both devices' probes simultaneously on the subcostal area of a patient while measuring DE could be a solution. Although we performed detailed statistical analyses to evaluate the correlations between measurements done with both devices, we also wanted to detect their performance in diagnosing DD in our study population. Despite lack of consensus about the definition of DD, in general, DD is accepted if TF is less than 20% and if its' tidal excursion is less than 10 mm [21, 24, 26]. When we consider these numbers as a cutoff point for DD, PSD could detect these patients as close as SDs in 36% of the patients. Lastly, it should be considered that different results reported in the literature can also be explained by the existence of technical differences between devices. Their brand, model, and capacity may be substantially different. While evaluating the performance of an ultrasound device, this point should be kept in mind. For example, the device we used had a dual probe which allowed us to evaluate not only DT but also DE easily. We used linear side of the dual probe for evaluating DT and sector side for the evaluation of DE. A PSD may also have MM depending on its technical properties. According to our results, weaknesses of a PSD are image acquisition difficulties in obese and edematous patients, difficulty in measurements and longer measurement times, and underestimation of maximal DE. In our study, the only parameter that was measured differently by two machines was maximal excursion of the diaphragm. In general, in the busy environment of an ICU, problems related with diaphragm rarely come across to mind. Widespread use of DUS may increase the diagnosis of respiratory problems caused by diaphragm pathologies in ICUs. Evaluation and monitoring of diaphragm function routinely in medical and surgical ICUs may also contribute to treatment decisions. DUS with PSD is immediately available, easy to learn, quick to perform, and applicable in a wide range of patients [23, 27]. In addition, it is possible to do the evaluation of many other internal organs within few minutes together with diaphragm. Furthermore, studies have been showing that they have good intra- and interobserver reproducibility. These results suggest that, in existence of limited time and resource settings, PSD could be as useful as standard USG devices for evaluation of the diaphragm.

## 6. Conclusion

Our results showed that PSD can visualize diaphragm in most of the ICU patients and can measure its thickness and excursion nearly close to standard machines. Rarely, they may fail in image acquisition in obese and edematous patients, and measurements are more difficult than the standard devices. Further studies are necessary to confirm these results and to determine the exact place of these devices in diaphragm evaluation.

## Figures and Tables

**Table 1:** Demographics of the patients.

*N* = 39	
Gender, F/M (*n*)	18/21
Age (yrs)	66 ± 18
BMI (kg/m^2^)	28 ± 6
APACHE-II	29 ± 7
Mechanical ventilation, *n* (%)	19 (48%)
Noninvasive mechanical ventilation, *n* (%)	10 (26%)
No ventilation, *n* (%)	10 (26%)
Admission diagnosis	
Pulmonary, *n*	38
COPD attack, *n*	21
Cardiac, *n*	20
Sepsis, *n*	15
Renal, *n*	14
Neurologic, *n*	7
Gastroenterologic, *n*	4
Endocrine metabolic, *n*	4
Mortality	10 (26%)

*N*, *n*: number; F: female; M: male; yrs: years; BMI: Body Mass Index; kg/m^2^: kilogram per square meter; APACHE-II: Acute Physiology and Chronic Health Evaluation Score.

**Table 2:** Diaphragm measurements of the patients with both devices.

	SD (mean ± SD) (min-max)	PSD (mean ± SD) (min-max)	*p*	ICC^∗∗^
Tidal expiratory thickness (cm)	0.27 ± 0.08	0.29 ± 0.11	^∗^	^∗∗^
	(0.13–0.46)	(0.13–0.61)		
Tidal inspiratoy thickness (cm)	0.37 ± 0.13	0.38 ± 0.14	^∗^	^∗∗^
	(0.19–0.85)	(0.20–0.70)		
Maximal inspiratory thickness (cm)	0.47 ± 0.16	0.45 ± 0.12	^∗^	^∗∗^
	(0.23–0.68)	(0.24–0.91)		
Tidal thickening fraction (%)	33 ± 17	34 ± 14	^∗^	^∗∗^
	(3–77)	(10–59)		
Maximal thickening fraction, %	69 ± 38	65 ± 31	^∗^	^∗∗^
	(8–150)	(20–130)		
Tidal diaphragm excursion (cm)	1.76 ± 0.69	1.62 ± 0.70	^∗^	^∗∗^
	(0.58–3.30)	(0.50–3.00)		
Maximal diaphragm excursion (cm)	2.97 ± 1.18	2.67 ± 0.90	a	b
	(1.33–5.40)	(1.30–4.70)		

SD: standard deviation; cm: centimeter; min-max: minimum-maximum; ^∗^there were no significant proportional bias, and there was good agreement between the devices' measurements (a); ^∗∗^interobserver correlation coefficient > 0.9 and *p* < 0.05; a: *p* value > 0.05; b: *p* value < 0.05.
